# A Research Agenda for the Assessment and Management of Acute Behavioral Changes in Elderly Emergency Department Patients

**DOI:** 10.5811/westjem.2019.1.39262

**Published:** 2019-02-19

**Authors:** Christina Shenvi, Michael P. Wilson, Alessandra Aldai, David Pepper, Michael Gerardi

**Affiliations:** * University of North Carolina, Department of Emergency Medicine, Chapel Hill, North Carolina; † University of Arkansas for Medical Sciences, Department of Emergency Medicine, Little Rock, Arkansas; ‡ University of California, San Diego Medical Center, Department of Emergency Medicine San Diego, California; § Hartford Hospital/Institute of Living, Department of Psychiatry, Hartford, Connecticut; ¶ Morristown Medical Center, Department of Emergency Medicine, Morristown, New Jersey

## Abstract

**Introduction:**

Agitation, mental illness, and delirium are common reasons for older adults to seek care in the emergency department (ED). There are significant knowledge gaps in understanding how to best screen older adults for these conditions and how to manage them. In addition, in areas where research has been performed, implementation has been slow. A working group convened to develop a set of high-priority research questions that would advance the understanding of optimal management of older adults with acute behavioral changes in the ED. This manuscript is the product of a breakout session on “Special Populations: Agitation in the Elderly” from the 2016 Coalition on Psychiatric Emergencies’ first Research Consensus Conference on Acute Mental Illness.

**Methods:**

Participants were identified with expertise in emergency medicine (EM), geriatric EM, and psychiatry. Background literature reviews were performed prior to the in-person meeting in four key areas: delirium; dementia; substance abuse or withdrawal; and mental illness in older adults. Input was solicited from all participants during the meeting, and questions were iteratively focused and revised, voted on, and ranked by importance.

**Results:**

Fourteen questions were identified by the group with high consensus for their importance related to the care of older adults with agitation in the ED. The questions were grouped into three topic areas: screening and identification; management strategies; and the approach to delirium.

**Conclusion:**

It is important for emergency physicians to recognize the spectrum of underlying causes of behavioral changes, have the tools to screen older adults for those causes, and employ methods to treat the underlying causes and ameliorate their symptoms. Answers to the identified research questions have great potential to improve the care of older adults presenting with behavioral changes.

## INTRODUCTION

Older adults, age 65 and over, account for approximately 15% of visits to emergency departments (ED) in the United States (U.S.).^1^ However, with the aging population, this is expected to increase to 25% by the year 2030.^2^ For many conditions, older adults are more likely to be misdiagnosed, have delayed diagnoses, and to have complications from their medical management.^3^ After an ED visit they are more likely to have functional decline, medical complications, or a revisit, re-hospitalization, or death.^4^ This is in part because of physiologic changes that occur with aging, underlying frailty or other geriatric syndromes, more medical co-morbidities, atypical presentations of symptoms, reduced physiologic reserve, and greater risk of medication complications or interactions. As a result, they are the population most at risk for decompensation if not identified and managed early.

Behavioral changes in older adults can arise from a range of different underlying causes, including delirium from an acute medical problem, dementia, alcohol or substance use or withdrawal, and mental illness. There are several barriers to the identification of causes of behavioral changes in older adults. One is that underlying dementia can make it difficult to obtain an adequate history. In addition, in the absence of collateral information, it may be impossible to determine whether there has been a change from their baseline. Delirium can also cause behavioral changes, but symptoms may wax and wane, and may present as hyper-active, hypo-active, or mixed. Despite the existence of ED-validated, rapid screening tools, emergency physicians find recognizing delirium in the majority of their patients challenging.^5^ It has also been shown that alcohol and substance use or withdrawal in older adults is under-recognized by physicians, which could lead to a delay in diagnosis.^6^ Finally, hearing and vision impairments in older adults can make it difficult for physicians to obtain an accurate history and physical exam. All of these challenges can impede the rapid identification of the causes of behavioral changes in older adults.

There are also challenges when it comes to management of older adults with behavioral changes. Dosages of psychoactive medications used in younger adults are more likely to result in side effects or sedation when used in older adults.^7^ Older adults are also more likely to suffer medication interactions, since on average they take more daily prescribed medications. Pharmacological and non-pharmacological interventions that improve outcomes of agitation or delirium in the ED have not been well studied.

Given the underlying medical complexity and frailty of older adults, the causes of their behavioral changes are both likely to be misdiagnosed, while they are potentially the most likely to benefit from early intervention. For these reasons, the research questions identified here have the potential to impact a significant number of vulnerable older adults in the ED.

## METHODS

Participants from a variety of disciplines – emergency medicine (EM), emergency psychiatry, emergency psychology, clinical research, governmental agencies, and patient advocacy groups – were invited to participate in a research consensus session held prior to a joint emergency-psychiatry conference (the 7^th^ Annual National Update on Behavioral Emergencies). Background literature reviews were performed prior to the in-person meeting. Literature reviews were conducted via journal review, academic databases, and web-based searches. Searches fell within the scope of the priority domain, geriatric behavioral emergencies, as identified by the Coalition on Psychiatric Emergencies (CPE) steering committee. The workgroup leaders identified articles of importance within four key areas: delirium; dementia; psychiatric illness; and substance abuse in the elderly. Key articles in these areas were circulated electronically to the group to review in advance of the in-person meeting. A nominal group technique was employed to develop group consensus on the highest priority research gaps. Following the nominal technique, input was solicited from all participants during the meeting, questions were iteratively focused and revised, voted on, and then ranked by importance. See Executive Summary and Methodology for full methods [Appendix].

## RESULTS

Key research questions identified by the multi-disciplinary working group were sorted into three categories: screening and identification; management strategies; and the approach to delirium. The working group was composed of eight individuals. There were two clinician emergency physicians (EP), one emergency clinician-researcher, two psychiatrists, and a non-physician student. The group also included two observers, one from industry and the other from an EM professional association. The average age of the participants was around 40 years old.

The group discussed the 37 articles that were reviewed in advance of the consensus conference. The working group identified 25 initial research questions to address gaps in the current literature. Using the nominal group technique the group then ranked the questions to identify the ones of most importance. Specific research ideas, questions and question variants were voted on using the dot method. Each participant was provided with 20 dots with which to vote. The questions that received four or more dots were considered more important. Those with three or less were considered less important. After voting, the group identified 14 questions that were considered of high importance for advancing the understanding of optimal management of older adults with acute behavioral changes in the ED. The questions were then discussed further, iteratively focused and revised. Following the in-person meeting, the workgroup developed additional consensus and worked electronically to further refine the final form of each question. Below we provide background information and a more detailed explanation for each question.

## DISCUSSION

The most important questions as identified by the workgroup are outlined below.

### Topic Area 1: Screening and Identification (Table 1)

#### Question 1: What are the barriers to screening for alcohol or substance abuse in older adults?

The ED represents an important point of contact during which alcohol use disorders (AUD) or substance use disorders (SUD) or high-risk use can be identified in patients who are asymptomatic or in those who present with behavioral changes from acute intoxication or withdrawal. AUDs and SUDs are prevalent yet under-recognized problems among older adults. The prevalence of AUDs among older adults is higher among patients within a healthcare setting compared with the overall prevalence in the community, with estimates of 14% for patients in ED, 18% for medical inpatients, and 23–44% for psychiatric inpatients.^8^ Many older ED patients with AUD may not be easily identified in the ED.^9^

The reasons for under-recognition of alcohol and substance use among older adults are likely multi-factorial. Elderly people may be less likely to disclose a history of excessive alcohol intake, and the problem is compounded by the fact that healthcare workers have a lower degree of suspicion when assessing older people.^10^–^11^ In addition, older adults may be unaware that their alcohol consumption is excessive or abusive until secondary events occur, and at that point may not attribute their problems to alcohol consumption.^12^

Another challenge to screening and identification of high-risk older adults occurs because many screening tools were developed and validated primarily in younger adults and may miss older adults. The Alcohol Use Disorder Test (AUDIT) and CAGE questionnaires have worse sensitivity and specificity among older adults using the traditional cutoffs, and do not perform well for the identification of high risk or heavy use.^13^,^14^ However, other screening tools have been developed specifically for older adults, such as the Short Michigan Alcoholism Screening Instrument-Geriatric Version, or the AUDIT score using a lower cutoff score.^15^

Once older adults with high-risk drinking are identified, they are less frequently referred for treatment. In one study, medical staff identified only 3% of benzodiazepine abusers, 38% of smokers, and 33% of drinkers. Of those identified, only 67%, 21%, and 58% patients, respectively, were referred for additional services.^16^ Among inpatients, older adults with alcohol use disorders are less often recognized and even when they are identified, they are referred for treatment at about half the rate of younger adults.^10^ This suggests that referral services are underutilized in this population, and medical staff may be biased against referring older patients.

Even though the American College of Emergency Physicians (ACEP) recommends routine screening and intervention in the ED for alcohol misuse,^17^ this practice has not been widely adopted in EDs for individuals of any age.^18^ Further research is needed on the best screening tools to identify AUDs and SUDs among older adults in the ED to discern the barriers to screening using existing tools or direct questioning of patients about alcohol intake, and to determine the most effective interventions after identification of high-risk patients.

#### Question 2: Using age as a stratification method, what are the medical and radiographic components of an appropriate medical screen for patients with psychiatric symptoms with an emphasis on sensitivity, specificity, and accuracy; do routine screening labs affect management and disposition in older adults with psychiatric symptoms?

Medical screening, commonly referred to as “medical clearance,” is a critical part of the ED evaluation of patients with mental health disorders, agitation, or behavioral changes.^19^ Specifically, medical screening is often required before a patient can be admitted or referred for admission to a psychiatric service or facility. Several studies primarily in younger patients have examined the medical screening of mental health patients.^20^–^21^ These studies have generally found that routine laboratory examinations are of low yield, prompting a recent ACEP task force to conclude that routine laboratory testing should not be ordered unless prompted by medical history, previous psychiatric diagnoses, or physician examination. However, this recommendation was given only a level C rating.^22^ In one retrospective study, authors subjectively determined whether abnormalities identified after admission would have changed management or disposition. In this report, the frequency of lab abnormalities was higher in patients over 40 years of age and almost universal in patients over 60 years of age. However, none of the abnormalities required transfer of a patient to a medical unit.^23^

Although general agreement exists that older psychiatric patients are at higher risk of medical disease, the exact age cutoff that would prompt routine screening is unknown. In addition, the optimal minimal screening studies required for these older patients are also not clear. There can sometimes be disagreement between EPs and psychiatrists as to what medical workup is required, such as whether imaging, routine toxicology, thyroid function tests, or liver function testing are necessary in all older patients. The more extensive workup may help identify medical pathology that is contributing to the psychiatric disorder. However, routine, extensive testing can also contribute to cost and length of stay (LOS).

While severe lab abnormalities identified on screening tests, such as severe hyponatremia, might warrant redirection from a psychiatric service to a medical service, it is not clear how often patients determined to have an acute psychiatric illness by the EP have significant incidental lab abnormalities on their screening tests. Further work is needed to make more concrete recommendations about medical screening tests needed in older adults presenting with psychiatric symptoms in the absence of other medical symptoms or complaints that would suggest a concurrent illness requiring medical management.

#### Question 3: How often does noncompliance with prescribed medications contribute to ED presentations with agitation or behavioral changes?

Older adults are prescribed more medications on average than younger adults. Particularly for psychiatric medications, accidental or intentional non-compliance on the part of the patient can result in acute behavioral or psychiatric symptoms. Among schizophrenic patients, non-compliance is thought to account for approximately 40% of return visits within two years of discharge and over $2 billion in readmission costs for this population alone.^24^ The scope of the problem in terms of how many visits for delirium, mental health, or acute agitation could have been prevented by improved medication compliance is not well defined. This is important to determine for several reasons. If non-compliance does account for a large percentage, then this would add evidence for the importance of a good medication history for older adults with behavioral changes. In addition, it could lend strength to interventions such as improved outpatient medication management strategies, proactive involvement of ED pharmacists, more thorough patient education about the risks of medication non-compliance, or systems to monitor medication use.

### Topic Area 2: Management Strategies (Table 2)

#### Question 4: What is the most effective pharmacologic agent to manage acute agitation in the acute care setting?

The symptoms of patients with delirium, behavioral changes, or acute mental health crises can sometimes not be managed solely through redirection or de-escalation. At times, psychotropic medications such as anti-psychotics or benzodiazepines are needed to maintain patient or staff safety or to treat the symptoms of agitation. The most effective medications for either treatment or prevention of delirium among older ED patients have not been well studied. Studies in the inpatient and post-surgical settings have not found a benefit from anti-psychotics for prophylaxis or treatment of delirium.^25^,^26^ Based on scant evidence, one recent expert consensus panel recommended that the underlying cause of the behavioral changes be treated first, and that medications be used as a second line, with low doses of second-generation antipsychotics being preferred in older patients only if necessary.^27^,^28^ In ED-based research, droperidol has been found to be safe and effective,^29^ but carries an FDA black-box warning about use in patients >65 years of age and is not available in many EDs. Intramuscular (IM) ziprasidone has also been studied among older adults with dementia.^30^ Of note, these medications are not without risks, and all antipsychotics are listed as potentially dangerous medications by the American Geriatrics Society Beers Criteria.^31^

Medications used to manage agitation may worsen other conditions, such as delirium, or cause gait instability. Efficacy must be weighed against side effects such as sedation, extra-pyramidal symptoms, and QT prolongation. The optimal dose and choice of medication for older patients will vary depending on the underlying etiology of their agitation and coexisting medical problems. The optimal medications, and their impact in terms of LOS and symptom severity and duration have not been well established.

#### Question 5: Does earlier treatment with psychotropic medications decrease length of stay in the ED for elderly agitated patients and does choice of treatment matter?

Decreasing LOS is also important to decrease ED crowding and potential adverse events.^32^ Some authors have noted that psychotropic medication administration may increase LOS for psychiatric patients compared to patients who do not receive medication.^33^,^34^ A retrospective study found that use of physical or chemical restraint in patients over 65 years of age was associated with longer LOS by over 12 hours.^35^ Patients requiring repeat doses of IM antipsychotics had a significantly longer LOS in the ED compared with non-repeat users of IM antipsychotics. However, patients who were initially administered oral, second-generation antipsychotics did not have longer stays in the ED even if a repeat dose was given.^36^ Given the association of many psychotropic medications with delirium and their sedating side effects it is plausible that medication choice in the ED may affect disposition or even cause a delay in discharge or admission.^37^ However, this has not been conclusively demonstrated in a prospective study.

#### Question 6: How often are older adults restrained physically or chemically in the ED; does the rate of restraint use vary with underlying known psychiatric disorders, and what are the potential harms or benefits of their use?

Approximately 10–30% of elderly patients in the ED have acute delirium,^38^ and it is often under-recognized and difficult to manage.^39^ There is also evidence that patients with psychiatric illness such as bipolar disorder,^40^ dementia, or depression are at greater risk for delirium.^41^ The use of physical or chemical restraints in the treatment of delirium has been studied in other settings such as skilled nursing facilities^42^ and intensive care units,^43^ and restraint prevention programs have been suggested.^44^ Several studies have shown that most patients will be cooperative with an oral dosing regimen despite the belief that they may be too agitated or uncooperative.^45^ An injected medication is likely to be experienced as assault rather than therapy or relief.^46^

Little work has been done to describe restraint use among older adults in the ED. The factors that predispose to restraint use, such as underlying psychiatric illness, or nature of the behavioral changes, physical strength, and other potential factors have not been defined. In addition, there are many potential risks and benefits of restraints in older adults, including potential harm to the patient with restraints, and potential patient or staff harm without restraints. Research into the outcomes of restraint use in this population would help better define the risks and benefits in order to aid providers in deciding on whether to use restraints, and which form to use.

#### Question 7: What are barriers to ordering pharmacologic treatment for acute psychiatric illness in the ED among older adults?

The use of any medication is more complicated among older adults due to their higher risk of adverse medication side effects or interactions with other medications. However, medications can also improve their symptom management and can generally be safely used among older adults. For example, the use of risperidone, ziprasidone, and olanzapine for treating acute agitation allows patients to follow oral maintenance treatment once the acute symptoms are ameliorated.^47^ In addition, many patients take benzodiazepines or anti-psychotics on a regular basis, and failure to give them their regular, scheduled dose could lead to the emergence of symptoms of their underlying disorder or withdrawal symptoms.

There are a number of potential barriers to treating older adults with psychiatric illness in the ED, including patient factors (unwillingness to take oral medications, difficulty providing a history, severe altered mental status or agitation) and provider factors (lack of knowledge of appropriate medications, concern about side effects, or failure to obtain a detailed medication history). As a result, there may be missed opportunities for better symptom control, which could lead to worse outcomes. The treatment of agitation and aggression needs to be further refined.^48^

#### Question 8: Does the initiation of home-based services for patients discharged from the ED with dementia help reduce the rate of ED return visits?

With an estimated 3.8 million Americans with dementia,^49^ proper treatment in the ED and on discharge from the ED is essential. Patients with dementia frequently present to the ED when they cannot be safely managed in their home environment, when they have been aggressive, have had medication complications, or have had frequent wandering and falls.^50^ Home health visits or other home-based services such as physical or occupational therapy, home physician visits, meal delivery services, or medication delivery services could potentially help prevent ED visits. Home-based care programs have been found to improve independence and quality of life for patients and caregivers.^51^ Patients with dementia who have presented to the ED at least once may represent a high-risk cohort who are more likely to require additional ED-based care in the future.^52^ It is possible that intervening with this group could reduce future visits by improving medication compliance, health quality, and allowing medical problems to be managed at home. EDs have traditionally not been well equipped to arrange home healthcare services. However, initiating the orders for home-based services from the ED could potentially reduce ED recidivism among high utilizers.

#### Question 9: What are the necessary components of an effective decision-support tool to determine whether it is safe to start or stop psychiatric medications, and does the use of such a tool improve outcomes?

The initiation or discontinuation of psychiatric medications is a complex decision, requiring knowledge of appropriate indications for use of medications; which patients can safely take them given their history, comorbidities, and other medications; starting doses; which medications can be safely stopped; and which need to be tapered. It is estimated that 60%–83% of patients are taking antipsychotics for non-U.S. Food and Drug Administration-approved conditions, with an estimated cost of $6.0 billion in 2008.^53^,^54^ Electronic prescribing devices with decision support systems significantly reduce error rates.^55^ However, such systems are costly and not widely implemented. Moreover, the use of electronic health records for decision support at the clinical level is not widely reported.

Given the complexity of the decision to start, stop, or alter the dose of psychiatric medication, physicians may benefit from decision support tools. Tools could search for interactions with other medications or provide guidance regarding indications, appropriate geriatric dosing, or the appropriate start and stop tapering time- frames. Decision support tools for this specific indication have not been well studied or widely implemented. Studies would need to show their impact and effect on clinical outcomes in order to provide support for their widespread use.

### Topic Area 3: The ED Approach to Delirium (Table 3)

#### Question 10: What are the barriers to diagnosis of delirium in the ED, and how can they be overcome?

Delirium is common among older adults in the ED and is associated with many adverse outcomes.^56^ Unfortunately, while common, it is also widely under-recognized. Some studies have reported it is missed approximately 57–83% of the time.^5^ Well-validated screening tools such as the Brief Confusion Assessment Method,^5^ and the Richmond Agitation Sedation Scale^57^ exist and have been studied specifically in older ED patients. Despite the existence of good screening tools, recognition of delirium remains low. This may be in part due to the heterogeneity of delirium, which can variably involve hypoactive, hyperactive, or mixed states, with waxing and waning severity. It may also be due to a lack of awareness of the importance of delirium in older adults, and lack of training in the available screening tools to identify delirium in these patients.

There are a mixture of patient factors (mixed presentation, hearing impairment, cognitive deficits, prior cerebrovascular accidents), provider factors (lack of awareness, lack of time), and systems factors (perceived lack of interventions if delirium is identified) that could contribute to the low rates of diagnosis. To increase the rates of recognition and eventually the early intervention for delirium, it must first be detected by the provider, nurse, or other member of the healthcare team. Identifying the reasons for low recognition is the first step toward improving identification and outcomes for patients with delirium in the ED.

#### Question 11: Is ED length of stay an independent risk factor for the development of delirium?

The ED, for many reasons, is a potentially deliriogenic environment; so longer LOS could lead to the development or worsening of delirium. ED boarding and crowding are a growing and multifactorial problem nationwide that can lead to prolonged ED LOS.^58^ Prior studies have shown a higher risk for delirium with ED LOS over 10 hours.^59^ Delirium predicts longer inpatient LOS^60^ and is an independent risk factor for six-month mortality.^61^ The association between ED LOS and the development of delirium has not been widely studied enough to generalize the findings. In addition, it is important to understand what factors about a prolonged ED LOS contribute to the onset of delirium in order to develop effective strategies or policies to intervene and prevent it.

#### Question 12: Does ED length of stay contribute to worse morbidity and mortality or adverse medical events in older adults with delirium?

It is known that longer ED LOS are associated with longer inpatient stays.^62^ In addition, delirium in older ED patients is an independent predictor of longer hospital LOS^60^ and six-month mortality.^61^ It is possible that longer ED LOS could cause higher rates of morbidity and mortality for delirious older patients. Older adults may have longer stays due to the need for more extensive testing, more complex disposition decisions, and the need to obtain collateral information. In addition, due to boarding and crowding, longer ED stays for all patients are becoming more common. It is therefore important to determine whether the longer stays are associated with higher rates of morbidity, mortality, or adverse events for patients with delirium.

#### Question 13: What are the most effective non-pharmacologic interventions in the ED to manage or prevent delirium?

Delirium occurs in about 20% of hospitalized older adults and 70–87% of older adults in the intensive care unit, and costs over $7 billion annually.^63^ Preventing delirium is the most effective strategy for reducing its complications, morbidity, mortality, and cost. Many multimodal, or multidisciplinary, non-pharmacologic interventions have been studied for delirium in the inpatient and post-operative settings. These may include early mobilization, fluid/electrolyte balance and hydration, frequent redirection, provision of activities, pain control, natural light during daylight hours, regulation of sleep/wake cycles, minimization of interruptions during sleep, proactive provision of hearing- and vision-aid devices, and minimization of psychoactive medications, among others.^64^,^65^

Protocols for reducing delirium among older ED patients have been suggested in nursing^66^ and EM literature.^67^ However, there have not been sufficient studies in the ED to determine and quantify what measures may reduce the rates of development of delirium among high-risk patients, improve the symptom severity of delirium, reduce the length of delirium, or reduce hospital LOS. Potential interventions would need to be feasible within the ED setting, cost effective, and easy to implement. Given the high cost as well as the long-term cognitive changes and increased mortality associated with delirium, this represents an extremely important question for the field of EM.

#### Question 14: Does having an ED pharmacist involved in patient care help reduce rates of delirium in the ED?

Many medications and combinations of medications commonly used in the ED can worsen or contribute to delirium in older adults. Delirium could be worsened by inappropriate medication selection or the use of doses that are too high for older adults. The involvement of ED pharmacists in patient care have been shown to help with accurate medication use and dosage, as well as improve time to appropriate treatment for time-sensitive conditions such as sepsis^68^,^69^ and stroke.^70^,^71^ Having an ED pharmacist review home medications for older patients in the ED with altered mental status or behavioral changes could help identify causes of delirium. In addition, an ED pharmacist review of medications and doses administered within the ED could help reduce overmedication, which can cause or prolong delirium, or dangerous medication combinations in delirious patients.

## LIMITATIONS

There are several limitations to this study. First, this was not an empirical review of literature, but rather an expert consensus group, which was held in 2016. While individuals with expertise in the care of older adults in the acute care setting were integral to the discussion, there were no internal medicine-trained geriatricians in the consensus group. By the time this paper is published, it is possible studies will have been conducted that answer or speak to some of the highlighted questions raised.

Second, the group focus was narrowed to four key areas: delirium; dementia; substance use or withdrawal; and mental illness in older adults. It did not focus on the less common reasons that older adults present to the ED with acute brain dysfunction or altered mental status such as neurologic diagnosis, including stroke or intracranial hemorrhage. The group felt it was of greater impact to focus on the more common medical and psychiatric reasons older adults present to the ED with confusion or agitation.

## CONCLUSION

Older adults represent a growing proportion of the population and account for a disproportionately high number of ED visits. There are numerous, multifactorial challenges that can make the screening, assessment, and management of behavioral changes more difficult compared with younger adults. Consensus building and discussion among a diverse set of stakeholders should be a priority for future research. In addition, there are significant knowledge and implementation gaps. The topics discussed here represent critical research questions to move the field forward and help emergency physicians provide better care to older adults presenting with agitation or behavioral changes.

To address these knowledge and implementation gaps, further research is needed in the key areas identified here. Successfully addressing these challenges will require research involving interprofessional teams as well as a public health perspective. Many of the solutions are beyond the scope of an individual clinician’s capabilities. The solutions will require systems-based or hospital-based changes and integration with other teams, such as social work, nursing, pharmacy, and outpatient or home-based care. Because of the integrated nature of high quality care of geriatric patients, the research will also need to involve interprofessional teams to be successful.

The prioritization of research questions in the area of geriatric behavioral health emergencies will help guide future research to solutions that have the potential to improve the care of older adults presenting to EDs with behavioral changes.

## Supplementary Material



## Figures and Tables

**Table 1:** Key research questions to guide efforts for improved care of older adults with behavioral changes through screening and identification.

Question 1	What are the barriers to screening for alcohol or substance abuse in older adults?
Question 2	Using age as a stratification method, what are the medical and radiographic components of an appropriate medical screen for patients with psychiatric symptoms with an emphasis on sensitivity, specificity, and accuracy; do routine screening labs, including urine, affect management and disposition in older adults with psychiatric symptoms?
Question 3	How often does noncompliance with prescribed medications contribute to emergency department presentations with agitation or behavioral changes?

**Table 2:** Key research questions to guide efforts for improved care of older adults with behavioral changes through improved management strategies.

Question 4	What is the most effective pharmacologic agent to manage acute agitation in the acute care setting?
Question 5	Does earlier treatment with psychotropic medications decrease length of stay in the ED for elderly agitated patients and does choice of treatment matter?
Question 6	How often are older adults restrained physically or chemically in the ED, does the rate of restraint use vary with underlying psychiatric disorders, and what are the harms or benefits of their use?
Question 7	What are barriers to initiating pharmacologic treatment for acute psychiatric illness in the ED among older adults?
Question 8	Does the initiation of home-based services for patients discharged from the ED with dementia help reduce the rate of ED return visits?
Question 9	What are the necessary components of an effective decision-support tool to determine whether it is safe to start or stop psychiatric medications, and does the use of such a tool improve outcomes?

*ED,* emergency department.

**Table 3:** Key research questions to guide efforts for improved care of older adults with behavioral changes through improved identification and management of delirium.

Question 10	What are the barriers to diagnosis of delirium in the ED, and how can they be overcome?
Question 11	Is ED length of stay an independent risk factor for the development of delirium?
Question 12	Does ED length of stay contribute to worse morbidity and mortality or adverse medical events in older adults with delirium?
Question 13	What are the most effective non-pharmacologic interventions in the ED to manage or prevent delirium?
Question 14	Does having an ED pharmacist involved in patient care help reduce rates of delirium in the ED?

*ED,* emergency department.
